# Editorial: Tick Saliva: Secret to Blood Feeding Success

**DOI:** 10.3389/fcimb.2022.885240

**Published:** 2022-03-17

**Authors:** Daniel E. Sonenshine, Patricia A. Nuttall, Sukanya Narasimhan

**Affiliations:** ^1^Department of Biological Sciences, Old Dominion University, Norfolk, VI, United States; ^2^Department of Zoology, University of Oxford, Oxford, England, United Kingdom; ^3^Department of Internal Medicine, Yale School of Medicine, Yale University, New Haven, CT, United States

**Keywords:** ticks, saliva, immunosuppression, blood feeding, cement, evasins

This special issue comprises new and recent research that offers fresh insights into the complex molecular organization of tick saliva that enables these ectoparasites to consume huge quantities of blood. Ticks are foremost amongst all blood-feeding arthropods in the ability of their saliva to minimize their host’s recognition of the skin lesion, dilate blood vessels, prevent blood coagulation and, in ixodid ticks, remain attached for days or weeks. In ixodid ticks, the salivary glands also secrete cement, facilitating long-term feeding while also excreting excess blood meal water, enabling them to greatly increase blood consumption. Although a great deal is known about tick saliva and many of its numerous molecules, the story is still incomplete. Precisely how ticks accomplish these remarkable feats, and the pathogens that exploit these processes, are explored in the scientific articles included in this special Research Topic. The 6 articles of this special issue present some of the latest discoveries and insights into how tick saliva manipulates the vector-host interface to benefit the tick and facilitate tick-borne pathogen transmission.

In a novel study addressing phosphorylation of salivary proteins, Agwunobi et al. show the remarkable importance of this relatively understudied group of salivary proteins. The authors found that they play a decisive role in regulating vital intracellular and even extracellular transport processes, cell adhesion, and cellular and metabolic processes in the host tissues. Using RNAi to silence expression of representative proteins involved in phosphorylation, they showed that these proteins are essential for normal blood feeding and reproduction.

A new *in silico* study (Bhattacharya and Nuttall) of tick salivary transcriptomes identified 399 transcripts related to the 50 previously characterized chemokine-binding proteins known as evasins. The biochemically characterized evasins from ixodid tick species are separated into two functional classes with exclusive binding to either CC- or CXC- chemokines. They appear to neutralize host chemokine function and hence suppress inflammation.

Phylogenetic analysis of the evasins together with the newly identified evasin-like proteins revealed two classes of CC-binding proteins, A1 and A2, with A1 exclusive to Metastriate species (*Amblyomma*, *Dermacentor*, *Hyalomma* and *Rhipicephalus*) and A2 exclusive to Prostriate species (*Ixodes*). CXC-binding proteins (class B) were found in both Prostriate and Metastriate species. It remains to be determined whether all these evasin-like proteins are functional and bind chemokines or have other roles in modulating the host response to ixodid tick feeding.

To understand how tick immunomodulatory proteins, e.g., evasins, serpins, cystatins, etc. interact with host anti-tick defenses, Denisov and Dijkgraaf review structural analyses of their folding patterns. Knowledge of the intricate details of these folds, including α-helices, β- strands, N-loops, etc., as well as the number and location of cysteines that form disulfide bonds, are described. Analyses of folding patterns help reveal the binding sites where parts of these molecules bind to host defense compounds and disable their activity. Descriptions of chemokine- binders (evasins), serine protease inhibitors (serpins), cysteine protease inhibitors (cystatins), lipocalins (e.g., OmCI complement inhibitor), and lectin pathway inhibitors, are illustrated to show their structural folds and how they interact with the host’s substrate molecules to disable them and compromise their ability to prevent tick feeding.

Neelakanta and Sultana provide a review on how tick saliva disrupts the host’s hemostatic processes and how vertebrate hosts respond with potent anti-tick defenses. This article presents additional information about the roles of well-known salivary proteins, e.g., lipocalins, anti-coagulants, and cement proteins, but also describes new information about lesser- known proteins and peptides, e.g., heat shock proteins, organic anion transporting polypeptides, as well as extracellular vesicles and exosomes which contribute to modulating the tick bite lesion. Collectively, these salivary constituents suppress and/or evade host immune defenses, facilitating successful tick feeding and tick-borne pathogen transmission. The authors consider how research on tick salivary gland products and the emerging understanding of the tick microbiome may help develop anti-tick vaccines.

A novel approach to understanding networking of diverse salivary molecules to facilitate tick feeding is described by Fernández-Ruiz and Estrada-Peña. To enable a holistic view of sialome expression, the authors constructed a directed network in which the nodes are salivary proteins functioning as the sources with tick biological processes serving as the targets. They applied this novel construct to an ixodid tick, *Rhipicephalus sanguineus*, feeding at different time intervals. The approach was also applied to different organs of an argasid tick, *Ornithodoros rostratus.* Network output measured the connections between proteins and processes with a strength directly proportional to the transcripts per million reads for each protein. The authors suggest that these findings may offer a greater understanding of how the tick and host interact with each other temporally. This network approach targets a level above more familiar lists of salivary proteins and host responses, aiming to link families of proteins with the different biological processes that occur during the feeding period.

In this special issue, the final contribution is from Ali et al. and presents an updated review of the complex molecular mechanisms underlying the vertebrate host responses to diverse elements introduced in the saliva of biting ticks, and how they compromise these defenses to evade detection. They suggest that the sequential expression of the salivary proteome is reflecting an ongoing strategic “arms-race” with the host immune response and emphasize the need to understand the temporal dynamics of salivary proteomes functionally aligned with host defense responses. The authors argue that precise knowledge of the temporality of specific tick proteins in the context of host defense responses will help develop effective anti-tick vaccines.

Together, these articles highlight how advancements in protein, RNA and DNA sequencing technologies have catalyzed our understanding of the composition of tick saliva. We now have an abundance of omics data describing components of tick saliva, but we lack a functional understanding of most salivary molecules. Research emphasis must shift from “cataloging” to functional characterization. Developing robust and creative *in silico* analysis suites may be critical to gaining functional insights. Lack of molecular tools to genetically manipulate the tick genome remains a major bottleneck. While RNA interference technology has provided meaningful functional insights, it is severely limited in its inability to provide robust and sustained phenotypes. The adaptation of CRISPR technology (Nuss et al., 2021) to tick biology may offer the much-needed push into the tick functional genome.

## Author Contributions

All authors have contributed equally to the editorial statement and approved the submitted version.

## Conflict of Interest

The authors declare that the research was conducted in the absence of any commercial or financial relationships that could be construed as a potential conflict of interest.

## Publisher’s Note

All claims expressed in this article are solely those of the authors and do not necessarily represent those of their affiliated organizations, or those of the publisher, the editors and the reviewers. Any product that may be evaluated in this article, or claim that may be made by its manufacturer, is not guaranteed or endorsed by the publisher.
